# Application of artificial intelligence-based stemness index in cancer

**DOI:** 10.3389/fonc.2025.1608712

**Published:** 2025-08-13

**Authors:** Liyuan Liu, Qin Pei, Javeria Qadir, Yiyu Chen, Jingyuan Li, Yanan Luo, Jiawen Xian, Rongrong Du, Ting Ye

**Affiliations:** ^1^ Department of Laboratory Medicine, the Affiliated Hospital of Southwest Medical University, Luzhou, Sichuan, China; ^2^ Department of Biosciences, COMSATS University Islamabad, Islamabad, Pakistan

**Keywords:** cancer stem cells, artificial intelligence, MDNAsi, mRNAsi, stemness

## Abstract

Cancer stem cells (CSCs) exhibit self-renewal and multidirectional differentiation capacities. The stemness of CSCs is the fundamental cause of tumor progression and treatment resistance. The stemness index, evaluating the number and activity of CSCs, is a crucial indicator predicting various aspects of tumor behavior such as growth, metastasis, and prognosis. With the advancements in artificial intelligence (AI), particularly in data analysis and machine learning, the identification and understanding of CSCs’ stemness characteristics have improved. The AI-based analysis allows for processing vast datasets and recognizing patterns that assist in comprehending the role of CSCs in cancer development. The utilization of AI to analyze and compute the stemness index holds significant clinical relevance in tumor diagnosis and treatment. This approach provides more precise and personalized information, potentially influencing treatment strategies. Therefore, tailoring treatments specifically targeting CSCs is highly imperative and may enhance therapeutic efficacy and outcomes in cancer patients.

## 1 Introduction

Cancer stem cells (CSCs) refer to the existence of a small subset of cells within a tumor that exhibit stem cell-like properties. These CSCs possess the ability to self-renew and differentiate into various cell types within the tumor, contributing to tumor growth and heterogeneity. This property of self-renewal allows CSCs to sustain their population within the tumor, acting as a source of cells that propagate and regenerate the tumor mass. Furthermore, CSCs have the capacity to prompt multidirectional differentiation, thus, generating different cell types within the tumor, often referred to as cancer-initiating cells (1–3). CSCs exhibit characteristics encompassing self-renewal, multidirectional differentiation, multi-drug resistance and radiation resistance, and signaling pathways that are common to both tumor and normal stem cells (4–6), hence, rendering them highly drug-resistant, evading conventional treatment, and more prone to cancer relapse and metastasis. Comprehending the molecular regulation of CSCs self-renewal is crucial for advancing cancer biology research and revolutionizing cancer treatment. However, there are numerous challenges that impede extensive apprehension and effective targeting of CSCs in cancer. For instance, the absence of highly effective and specific techniques for CSCs identification and isolation presents a significant challenge, which may be attributed to their small subset representation within tumors (7). Additionally, the intricate molecular mechanisms governing CSCs self-renewal are not yet fully elucidated, thus, hampering the development of targeted therapies that specifically address CSCs populations (8). Therefore, a clearer understanding of CSCs and their role in driving cancer progression is necessary, as imprecise knowledge of the molecular underpinnings of CSCs behavior may obstruct the development of effective anti-cancer treatments (9). Moreover, advanced tools and breakthroughs are highly required in regards to CSCs isolation and characterization, that would facilitate efficient identification, isolating and assessment of CSCs, thus, propelling advancements in the niche of CSCs research (10). Regardingly, state of the art techniques such as single-cell sequencing, advanced imaging techniques, and more refined biomarker identification methods, may aid in optimum CSCs identification and characterization (11, 12). Moreover, collaborative efforts between researchers from various disciplines will be essential to unravel the complex nature of CSCs, thus, paving way for innovative clinical treatments targeting these cells. In 1994, Dick et al. used stem cell surface antigen labeling and flow cytometry for the first time to isolate and identify leukemic CSCs with stem cell markers (CD34+/CD38-) from human leukemia cells, which have potential to self-renew in acute myeloid leukemia (7).Subsequent studies used a similar approach to isolate and identify CSCs in different cancers such as breast (8), brain (9, 13), head and neck squamous cell carcinoma (10), pancreatic (11) and lung cancer (12) and prostate cancer (14). Most of the limitations of current methods for isolating and identifying cancer stem cells (CSCs) include insufficient precision, low yield of isolated cells (13), inability to mimic the complex tumor microenvironment (TME) (15), high-cost and procedural complexities, and inability to establish stable cellular models for subsequent future studies (16).

Integration of Artificial Intelligence (AI) in oncology has shown promising prospects in characterizing cancer stem cell properties, explicitly ‘stemness’. The complexity and high dimensionality of cancer-related data, including genomics, transcriptomics, and epigenomics, underscore the need for Artificial Intelligence (AI) in oncology (17). Traditional analytical methods often fall short in capturing intricate, nonlinear patterns within large datasets. AI, particularly machine learning and deep learning models, has enabled the identification of hidden biomarkers, classification of tumor subtypes, and prediction of treatment responses with improved accuracy. In cancer stem cell (CSC) biology, AI facilitates the integration of multi-omics data to define stemness signatures and identify rare CSC populations (18). It also aids in modeling CSC dynamics, predicting resistance mechanisms, and uncovering novel therapeutic targets. Clinically, AI is expected to support the development of personalized therapies by stratifying patients based on CSC-related risk and treatment sensitivity. As AI tools continue to evolve, they hold significant promise in bridging the gap between CSC research and real-world clinical applications (19). Rapid evolution of the high-throughput detection technologies entailing advanced imaging, genomic sequencing, and other -omics techniques, has enabled extensive data generation in regards to tumor histology (17, 18). This typically includes comprehensive tumor databases{TCGA(The cancer genome atlas), ICGC(International Cancer Genome Consortium), COSMIC, UCSC Cancer Genomics Browser, canEvolve, CGWB (Cancer Genome Workbench)}, Genome Database{Array Map, BioMuta, Cancer Hotspots, Mitelman Database, SomamiR, CGP (The Cancer Genome Project)}, DNA methylation database{MethHC, MethyCancer, MethDB, NGSmethDB, PubMeth, SurvivalMeth, DiseaseMeth2, MethSurv, MethBank, Lnc2Meth, MEXPRESS}, Transcriptome Database{Oncomine, GEO(Gene Expression Omnibus), ArrayExpress, ChiTaRS, miRCancer, OncomiRDB, UALCAN, CRN (Cancer RNA-Seq Nexus)}, Proteome. Database {Cancer3D, CancerPPD, Cancer Proteome Variation Database (CanProVar), Clinical Proteomic Tumor Analysis Consortium (CPTAC), DbDEPC}, Database of tumor-related genes {DriverDB, Network of Cancer Genes (NCG), TP53MULTLoad, UMDTP53} and oncology and drug databases {CancerDR, CancerResource, canSAR, Genomics of Drug Sensitivity in Cancer (GDSC), Platinum}. This synergy between AI technologies and high-throughput detection methods holds tremendous promise in advancing our understanding of CSCs and their role in cancer biology. The amalgamation of AI-driven technologies with tumor-related databases presents a plausible tool for uncovering characteristic gene expression patterns, signaling pathways, and molecular markers associated with CSCs, thus, enhancing our ability to comprehensively and systematically understand the stemness characteristics of (CSCs) (20, 21). Moreover, this would significantly advance both basic tumor research and subsequent clinical treatment strategies, hence, fueling discoveries that may be translated into practical applications, potentially revolutionizing cancer treatment and patient care.

This review presents a novel synthesis by integrating cancer stem cell (CSC) biology with emerging artificial intelligence (AI)-driven analytical approaches, offering a unique perspective on stemness indices across various cancers. While numerous studies have explored CSCs or AI separately, few have critically examined their intersection, particularly in the context of mRNAsi, mDNAsi, DMPsi, and ENHsi. By comparing methodologies, highlighting limitations, and evaluating translational potential, this review bridges a critical knowledge gap. Additionally, the compilation of databases and tools provides a practical guide for researchers, making this review both timely and valuable for advancing precision oncology and CSC-targeted therapeutic strategies.

## 2 Biological basis of stemness of CSCs

**Figure 1:**
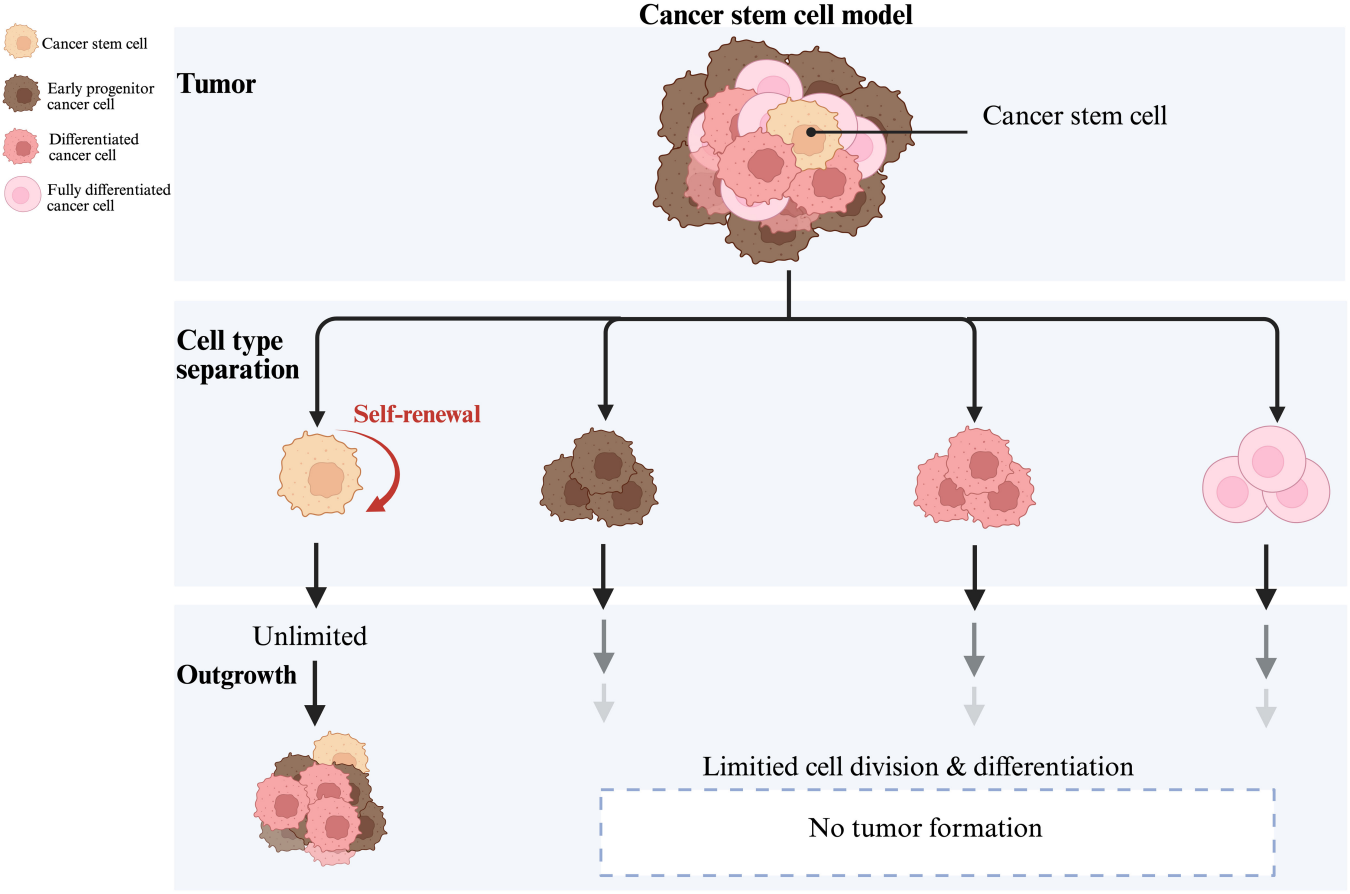
Symmetric and asymmetric division patterns of CSCs in tumor development.

Stem-like characteristics of CSCs include self-renewal capacity, differentiation potential, and high tumorigenicity (4, 5), as indicated in the **Figure 1**. Most distinguishing feature of CSCs is the capacity for self-renewal, generating specialized mitoses of one (asymmetric) or two (symmetric) daughter stem cells (4). When CSCs predominantly undergo symmetric divisions, they give rise to two identical CSCs, thus, culminating into an increased pool of CSCs within the tumor. Consequently, there is increased self-renewal capacity and an expansion of the stem cell population, contributing towards tumor aggressiveness, increased heterogeneity, and higher chances of malignancy. In contrast, asymmetric divisions generate one CSC and one differentiated progenitor or non-stem cell, maintaining a balance by replenishing the CSC pool while simultaneously producing cells that contribute to the tumor’s differentiated cell population, therefore, resulting in a more stable tumor phenotype (22). Once this balance between the symmetric and asymmetric CSCs divisions is attained, the tumor tends to stabilize; whereas, when the balance is tilted towards symmetric divisions, the proportion of CSCs increases and the tumor manifests itself as highly malignant (23).

**Figure 2:**
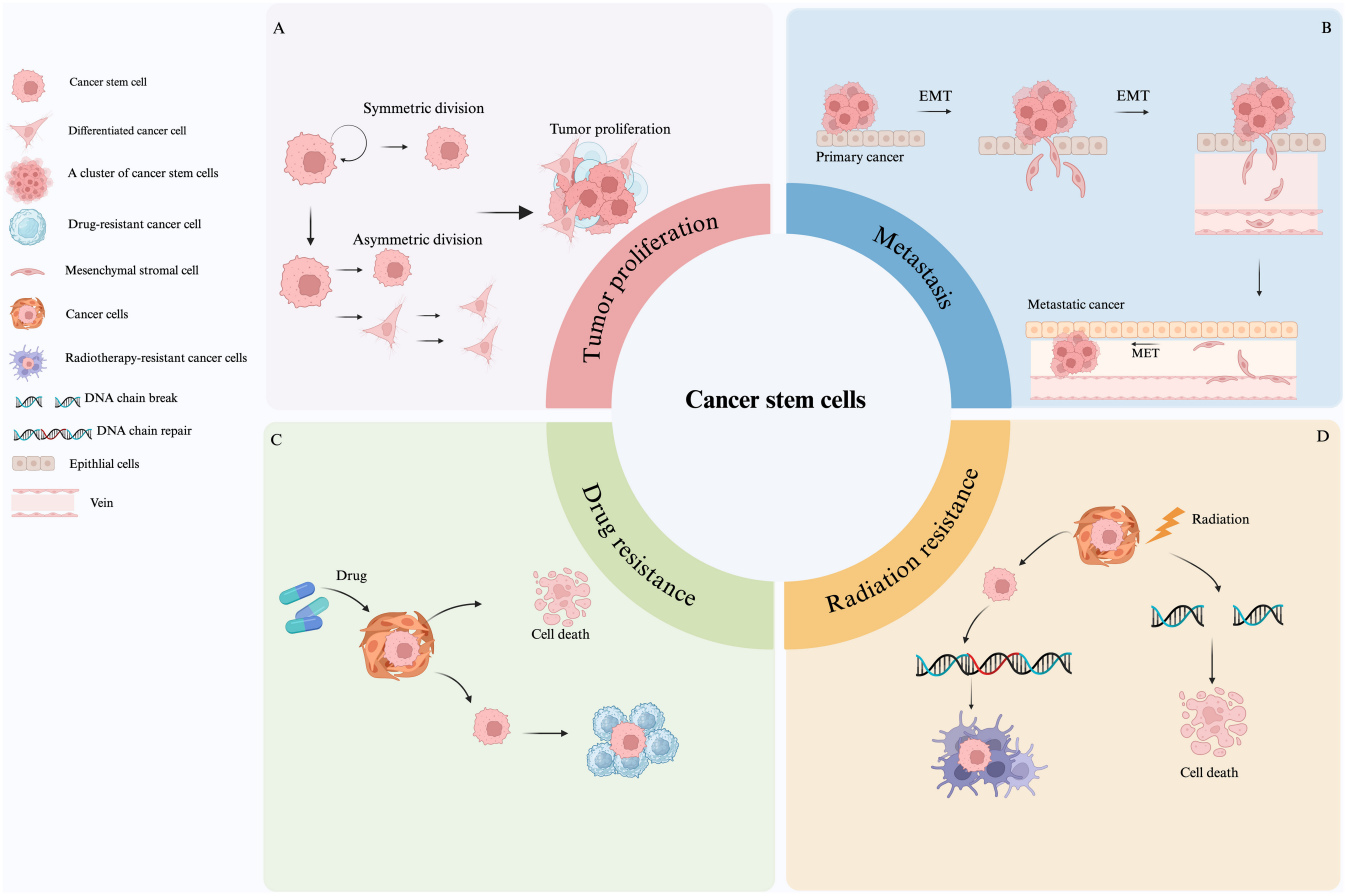
Roles of cancer stem cells in tumor proliferation, metastasis, and therapy resistance. **(A)** Mechanisms of CSC-driven tumor proliferation. **(B)** CSCs promotes metastasis primarily through EMT-related pathways. **(C)** CSCs express high levels of drug transporters, leading to chemoresistance. **(D)** CSCs resist radiation through enhanced DNA repair and microenvironmental protection.

Some researchers have begun working on developing methods aiming to control the malignant transformation of tumors using asymmetric division regulatory mechanisms (24). CSCs exhibit high stemness in a variety of tumors such as leukemia (25), breast (26), brain (9, 13), colon (27)and lung cancer (28), and are involved in tumor growth, maintenance, and progression. Furthermore, the plasticity of CSCs (29) suggests that tumor cells can activate the stemness of CSCs by dedifferentiating and obtaining specific stimuli, leading to tumor recurrence. Additionally, CSCs stemness has a significant impact on cancer initiation, proliferation, metastasis, and therapy resistance (30, 31). **Figure 2** provides a comprehensive overview of how these stemness characteristics contribute to these processes. Firstly, the stemness of CSCs affects the direction and difficulty of cancer initiation and influences augmented cellular carcinogenesis. It is closely related to cell proliferation, whereby, the stronger the stemness, the higher the proliferation ability, as shown in **Figure 2A**. Tumor proliferative capacity and malignancy can be influenced by genetic, epigenetic, and proliferative modes of division regulation in CSCs. Secondly, the stemness of CSCs is also closely related to the ability of tumor metastasis, and their self-renewal and differentiation are often accompanied by cell motility and migration, allowing tumor cells to metastasize (32, 33). **Figure 2B** shows that the stemness genes of cancer stem cells mainly affect cancer metastasis through epithelial-mesenchymal transition (EMT)-related pathways (34). Studies have shown that EMT and the metastatic process of CSCs are quite similar, and their genes and transcriptomes have great overlap, suggesting that EMT cells and primary CSCs may be highly overlapping concepts (34). Although the same genetic markers are found in metastatic EMT and CSCs models, CSCs with high metastatic potential may not be a completely new type of cancer cell, but rather a subtype of CSCs or the result of a cellular gene mutation (35). For instance, in D133+ pancreatic CSCs, the migratory ability of the cells with high expression of CXC-chemokine receptor 4 (CXCR4) is significantly higher than those cells with low expression. As well, the patients with a high proportion of CD133+ and CXCR4+ cells in the cancerous tissues have higher probability of cancer metastasis (36). Briefly, the higher the stemness, the stronger is the metastatic ability. Finally, the characteristics of CSCs are closely linked to tumor therapy resistance. Specifically, when the stemness of CSCs is in a dormant state, they are insensitive to external physicochemical factors that kill tumor cells and can evade treatment (37, 38), leading to therapy resistance (39). Mechanisms of resistance vary with treatment and primarily include high expression of drug transporters that assist in the transfer of intracellular toxic chemicals, as seen in **Figure 2C**. Additionally, CSCs possess strong DNA repair capacity, enabling them to resist radiation genome disruption (**Figure 2D**). Furthermore, CSCs recruit non-cancerous stem cells to form a protective microenvironment that further contributes to resistance for CSCs (40). Comprehensively, identification of the stemness features of CSCs is crucial to gain insight into the molecular mechanisms of tumor progression. These stemness features are not only relevant to tumor progression and therapeutic resistance, but also important for timely diagnosis, selection of the therapeutic strategies, and patient prognosis monitoring.

## 3 Methodology for assessment of stemness indices

**Figure 3:**
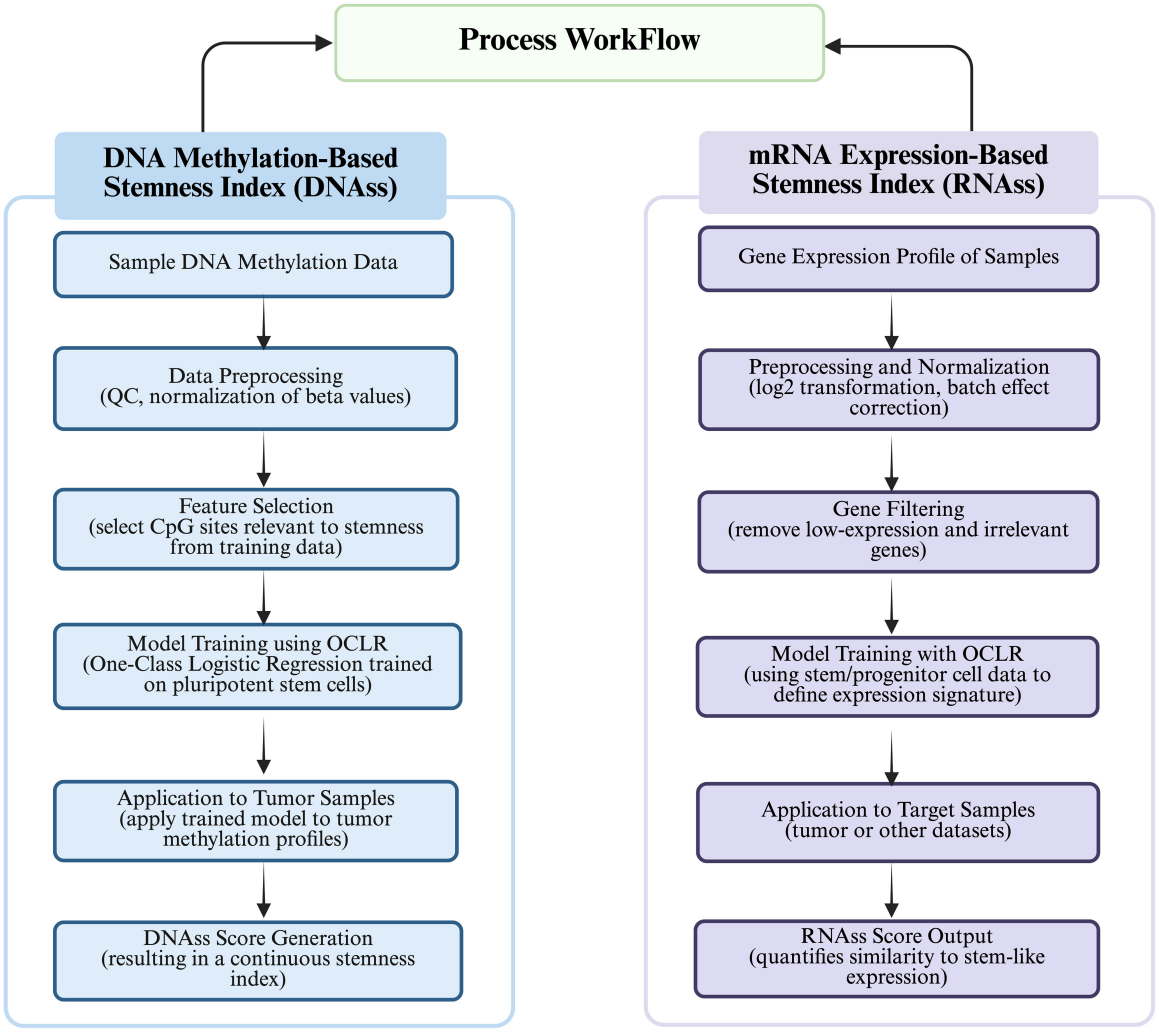
Process workflow depicting “Calculation of DNA methylation-based stemness index” and “Calculation of mRNA expression-based stemness index.”.

With the rapid development of gene sequencing technology and the improvements in data processing technologies, AI has become a hot spot in tumor research (41, 42). Of these, Network Analysis (NA) is an analytical method that uses genes or proteins as nodes and their interactions as edges (43). By constructing gene co-expression networks or protein interaction networks, a collection of genes related to a specific biological process, known as functional modules, can be identified (44), and ultimately the functional genes related to stemness can be identified by the relevance of these modules (45). Data mining through network analysis generates large-scale gene expression data, which can be utilized to identify stemness genes using machine learning with high accuracy and reliability (46). Currently, the methods employed for recognizing stemness genes based on machine learning algorithms are mainly categorized into (i) supervised learning, and (ii) unsupervised learning (47). One representative implementation of stemness index modeling is the work by Malta et al. (48), where the authors trained a one-class logistic regression (OCLR) model using stem cell-specific transcriptomic and epigenomic profiles obtained from the Progenitor Cell Biology Consortium (PCBC). The model was trained to capture the gene expression features of pluripotent stem cells and then applied to bulk tumor data from TCGA to generate a stemness index—referred to as (i) mRNAsi (based on transcriptome), derived via Spearman correlation between OCLR weights and tumor gene expression; (ii) mDNAsi (based on DNA methylation) (49), constructed by integrating three types of features: differentially methylated probes (DMPs) between stem cells and progenitors, methylation markers of stem cell-specific enhancers (via Roadmap Epigenomics ChromHMM data), and epigenetically regulated genes identified by ELMER, as comprehended in **Figure 3**. Following the establishment of stemness indices by Malta et al., subsequent studies—such as the development of the TS score in bladder urothelial carcinoma (BLCA)—have leveraged tumor stemness quantification to classify subtypes, predict prognosis, and assess immunotherapy responsiveness based on CSC and EMT features (50). These indices quantitatively reflect the similarity between a tumor sample and the stem-like transcriptional or epigenetic phenotype. Importantly, high mRNAsi scores and mDNAsi scores were found to correlate with dedifferentiation, poor prognosis, and therapy resistance across multiple cancer types, supporting the relevance of stemness-based metrics in oncology. Since then, many studies have been conducted to develop and refine stemness indices using machine learning algorithms and gene expression signatures (17, 50, 51) to assist in the prediction of tumor growth, metastasis, and prognostic information, which are clinically significant in tumor diagnosis and treatment (5, 24, 42).

## 4 Artificial intelligence driven stemness techniques in stemness analysis

### 4.1 Calculation of DNA methylation-based stemness index

**Figure 4:**
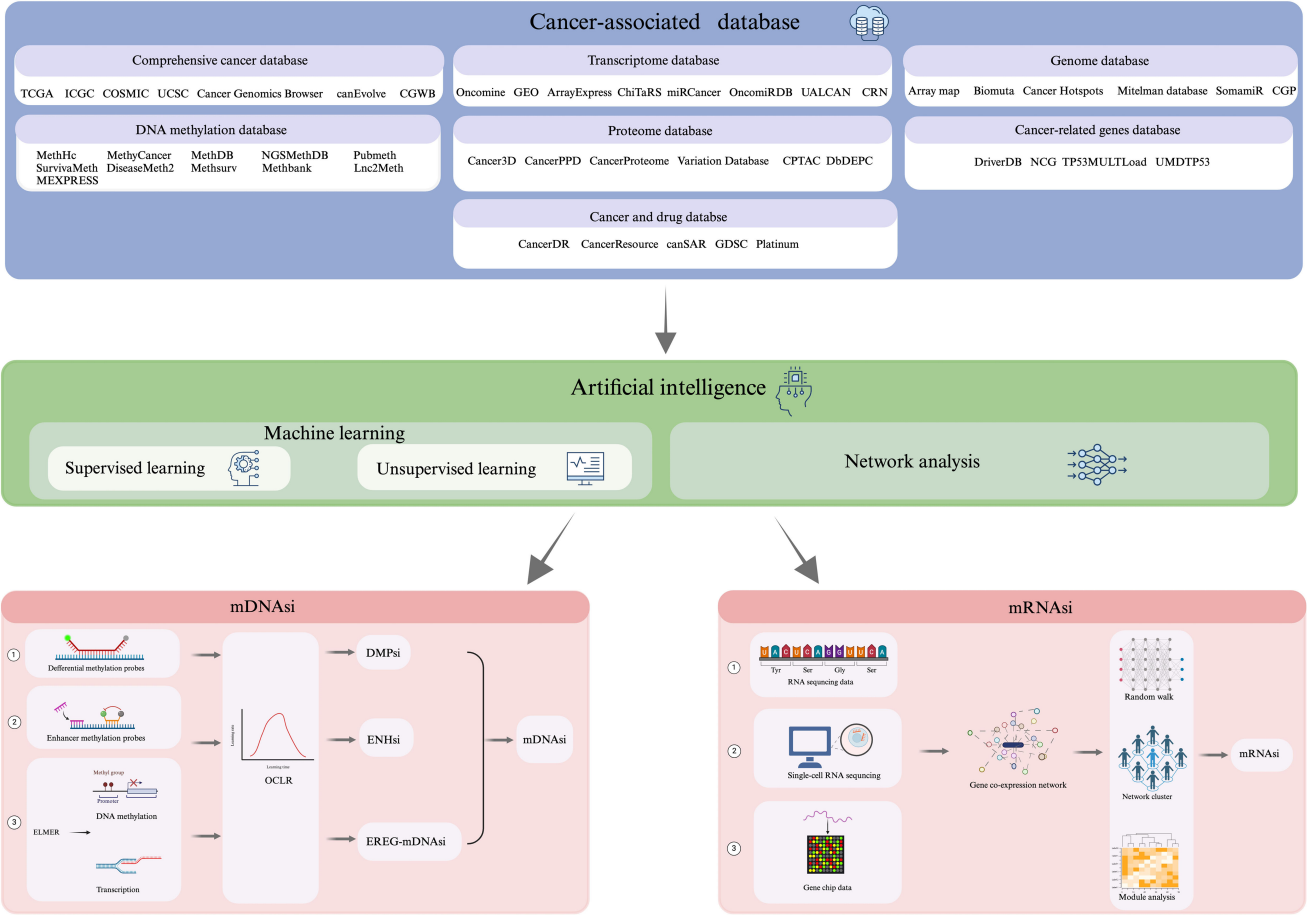
Calculation of the stemness index.

The process workflow for calculation of DNA Methylation-based Stemness Index has been illustrated in **Figure 4**. DNA methylation is the addition of methyl groups to cytosine residues in the DNA molecule, which regulates gene expression and cell differentiation, and is widespread in epigenetic modifications in eukaryotes, including CpG island methylation and non-CpG island methylation (52). During cell differentiation, stem cells usually have low levels of DNA methylation, whereas non-stem cells have high levels of DNA methylation (53). Malta (48) defined the mDNAsi using OCLR by combining: supervised classification between ESC/iPSC and their progenies, iPSCs and their progeny. Roadmap in the ChromHMM software as well as ELMER (Enhancer Linking by Methylation/Expression Relationships, ELMER) define mDNAsi on the basis of known methylation patterns of stemness and non-stemness genes using statistical models or machine learning algorithms to compute stemness indices including Euclidean distance, Pearson correlation coefficient and methylation clustering. The stemness index (SI) calculated from DNA methylation data involves the identification of specific CpG sites that exhibit differential methylation patterns between stem cells and non-stem cells. The differences in methylation levels at these CpG sites serve as a basis for quantifying the stemness of a particular cell sample or a tumor. The process typically involves identification of differential CpG Sites; calculation of Stemness Index; and quantification of Stemness (54). However, DNA methylation stemness features cannot be deciphered through a range of probes, however, different methylated regions are used as inputs, whereby, three methods are being employed depending on the input features (14, 48): (i) DMPsi (differentially methylated probes-based stemness index) uses differentially methylated probe regions (containing many filtering conditions) as input to the OCLR algorithm to construct predictive models; (ii) ENHsi (enhancer-based stemness index) uses methylated probes of enhancer regions as input to the OCLR algorithm to construct predictive models; and (iii) EREG-mDNAsi uses the ELMER package to reconstruct gene regulatory networks from DNA methylation and transcriptome expression data, and use the identified features as inputs to the OCLR algorithm to construct predictive models, which can generate methylated probes and genes as output. The DNA methylation-based approach for calculating stemness index consists of two steps (55): (i) Firstly, “stemness genes” specifically expressed in stem cells are identified based on Gene Set Enrichment Analysis (GSEA), Principal Component Analysis (PCA), and Machine Learning, and (ii) Secondly, the DNA methylation level of each CpG site in the promoter region is determined using microarray or sequencing-based methods to calculate the methylation score for each gene. Once the methylation levels are scored for each gene, the DNA methylation-based SI was calculated using the “EpiScore” algorithm (56), which identifies CpG sites that are specifically methylated in stem cells and also differentially methylated in cancer cells. Subsequently, the SI scores are used to determine the average methylation level of CpG sites within the stem cell-specific CpG set. For instance, Liu (57) et al. characterized copy number alteration and genome-wide DNA methylation of meningioma subtypes using random forests and constructed a meningioma progression score (MPscore) using the stemness index.

### 4.2 Calculation of mRNA expression-based stemness index

The process for calculation of mRNAsi has been illustrated in the **Figure 4**. The mRNAsi was calculated based on gene expression patterns, functional annotations, gene networks, and gene differential expression data while using statistical models or machine learning algorithms (58). The most commonly used data are single-cell RNA sequencing data, gene chip data, or RNA sequencing data (44). By using gene co-expression networks to analyze co-expression patterns between genes, functional annotation information, and gene or protein interaction networks, stemness indices are calculated using random walk algorithms, network clustering, and modular analysis (36). Pertinently, Malta (48) validated mRNAsi by applying it to an external dataset consisting of stem and somatic differentiated cells and scored the molecular subtypes of breast cancers and gliomas with higher SI values for all stem cell samples than for differentiated cell samples. Likewise, Tan (59) obtained potential molecular subtypes of GBM patients from the GlioVis dataset by using the Consensus Cluster Plus (CC) R package based on unsupervised clustering analysis into gene.clusters.C1 (C1), gene.clusters.C2 (C2) and gene. clusters.C3 (C3), followed by the analysis of the TME variants, immune cell infiltration, and stemness indices for the three subtypes. Similarly, Sun (60) used a non-negative matrix decomposition algorithm to efficiently reduce the dimensionality of the integrated dataset (an effective dimensionality reduction method widely used to differentiate molecular patterns in high-dimensional genomic data), classified the expression of anoikic-related genes into Cluster 1 and Cluster 2, and analyzed the differences between the two clusters in terms of TME, stemness indices, and clinical traits and constructed the risk-scoring model to evaluate the relationship between risk scores of glioblastoma and pan-cancers and the TME, stemness, clinical traits, and response to immunotherapy. Da (45) first used the hclust function to cluster the samples and remove the outliers, followed by the use of the soft Power = sft$powerEstimate command to select the optimal soft threshold to ensure that the interactions between lncRNAs conformed to the scale-free distribution to the greatest extent possible and constructed the neighbor-joining matrix by calculating the topological overlap matrix (TO). Afterwards, hierarchical clustering using (1-TO) was used as the distance metric, selecting key modules by identifying them through the dynamic shear tree algorithm, defining the lncRNAs in the key modules as stemness index-associated lncRNAs, and finally constructing stemness index-associated lncRNA markers for predicting prognosis in breast cancer patients. Li (61) et al. used a one-class logistic regression machine learning algorithm (OCLR) to extract the transcriptomic and epigenetic feature sets derived from untransformed pluripotent stem cells and their differentiated progeny to calculate the mRNAsi values (mRNAsi ranges from 0 to 1, the closer the mRNAsi is to 1, the stronger the stem cells’ features are), and found that the distribution of immune cells differed significantly between high and low mRNAsi lung cancer subtypes. Additionally, **Table 1** summarizes the characteristics of four commonly used stemness indices—mRNAsi, mDNAsi, DMPsi, and ENHsi—highlighting their underlying molecular basis, data sources, strengths, limitations, and typical applications. mRNAsi is based on gene expression data and reflects transcriptional activity, while mDNAsi utilizes genome-wide DNA methylation patterns to capture epigenetic regulation. DMPsi refines mDNAsi by focusing on differentially methylated positions, thereby improving specificity in stemness evaluation. ENHsi further emphasizes enhancer-associated methylation, offering insights into non-coding regulatory mechanisms. Each index provides a distinct perspective on tumor stemness, complementing one another in cancer classification, prognosis prediction, and the study of epigenetic plasticity.

**Table 1:** Comparative analysis of common stemness indic.

Stemness index	Basis	Data source	Advantages	Limitations	Applications
mRNAsi	Gene expression (mRNA)	TCGA, GEO (bulk RNA-seq)	Widely used; captures transcriptional activity; applicable across cancers	May miss epigenetic regulation; sensitive to tumor heterogeneity	Identifying stem-like phenotypes; correlating with prognosis and immune infiltration
mDNAsi	DNA methylation patterns	TCGA, GEO (450K/850K arrays)	Reflects epigenetic regulation; more stable than RNA-based indices	Limited to methylation sites; may not fully capture transcriptional dynamics	Cancer classification; predicting therapy resistance and epigenetic reprogramming
DMPsi	Differentially methylated positions	TCGA, GEO	Focuses on informative CpG sites; increased specificity	Dependent on high-quality DMP identification; limited cross-cohort comparability	Distinguishing tumor subtypes with differential stemness
ENHsi	Enhancer-associated DNA methylation	ENCODE, TCGA	Targets regulatory regions; reflects enhancer activity driving stemness	Complex analysis pipeline; enhancer regions less annotated in some cancers	Understanding non-coding regulatory influence on stemness and cancer progression

## 5 Clinical applications of AI-based stemness indices in cancer

The Stemness Index (SI) has gained significant importance in cancer research as a valuable metric for quantifying the extent of stem cell-like characteristics within a tumor cell population (62). By examining gene expression patterns and identifying genes that are commonly expressed in stem cells, the stem cell index can be used to predict tumor aggressiveness, patient prognosis, and response to therapy (63), providing new targets and strategies for cancer management (50).

### 5.1 Stemness index in cancer therapy response

**Figure 5:**
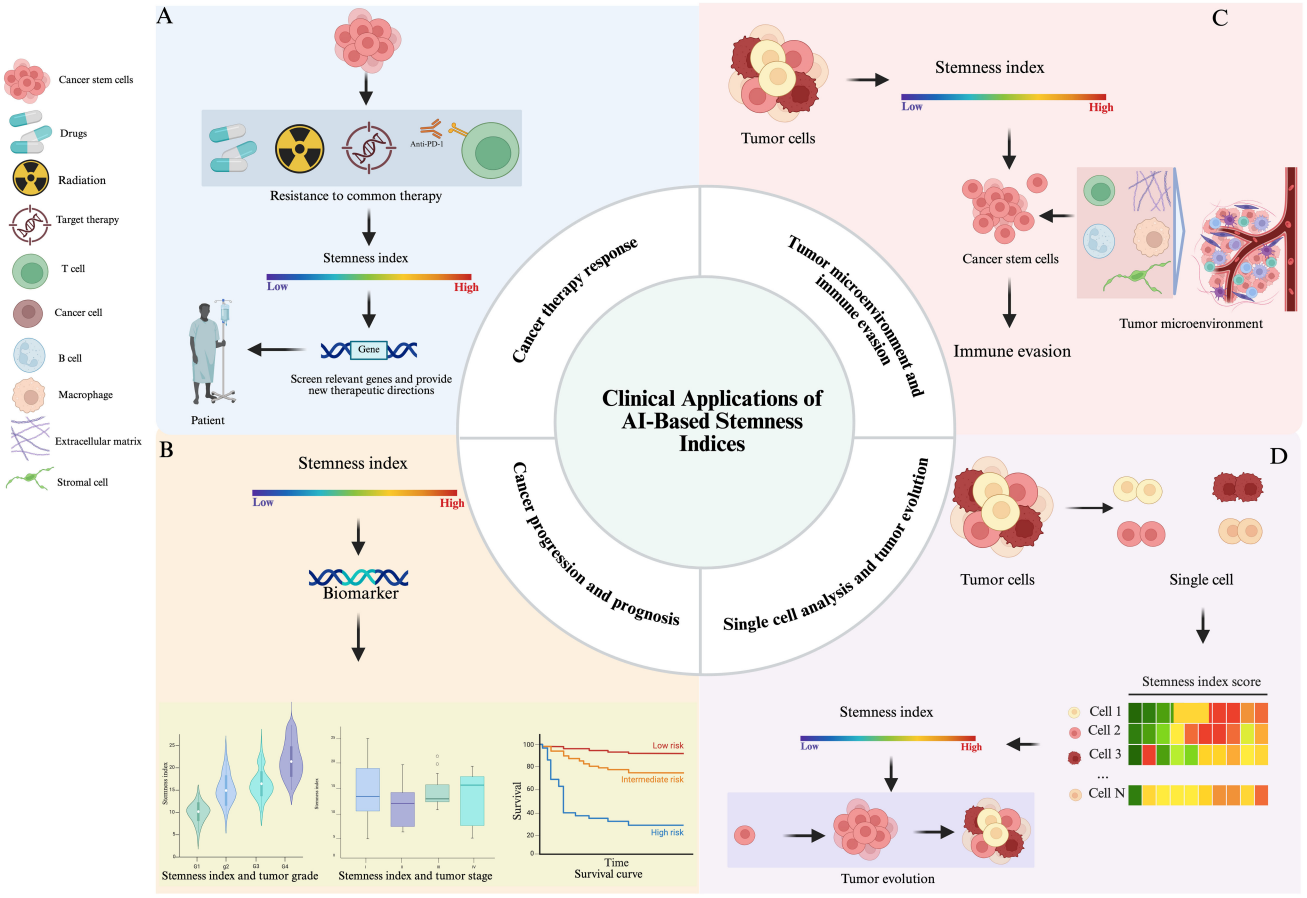
The clinical applications of AI-based stemness indices in oncology **(A)** Clinical application of stemness index in CSCs therapy resistance. **(B)** Stemness index as a potential biomarker for tumor grading, staging, and predicting prognosis. **(C)** The roles of stemness index in TME and Immune Evasion. **(D)** In single-cell analysis, the stemness index is helpful for identifying tumor subpopulations within heterogeneous tissues.

Currently, common treatment modalities for cancer include traditional radiotherapy, targeted therapy, and immunotherapy (42). The stem cell index identifies patients with tumors that have high levels of stem cells, reduces the number of patients who develop resistance to traditional chemotherapy and radiation, provides targeted therapies against CSCs, and improves treatment response rates (**Figure 5A**). CSCs are thought to be responsible for tumorigenesis, progression, and recurrence, and targeting these cells may improve overall therapeutic outcomes in cancer patients (42). In this regard, Guo (64) et al., screened 16 genes related to stem cell characteristics of IGC and 43 genes of DGC using mRNAsi. They preliminarily analyzed the relationship between the clinical features of gastric adenocarcinomas and the mRNAsi scores, and found that the tumor samples had higher stemness indices than the normal samples, whereby, there was a significant difference between intestinal-type and diffuse-type gastric carcinomas; and that the stemness-properties-related genes were related to the cell cycle, and they could be used as a therapeutic target for inhibiting the stem cells of gastric cancer.

Furthermore, using the mRNAsi from The Cancer Genome Atlas (TCGA) to assess and correct tumor purity, along with the exploration of gene modules and key genes through weighted gene co-expression network analysis (WGCNA), has shown that grade III and IV tumors have higher mRNAsi and corrected mRNAsi scores than grade I and II tumors (65). This research verified the expression of 13 key genes between advanced platinum-resistant and sensitive SOC samples in two Gene Expression Omnibus (GEO) datasets, which showed CDC20 to be a potential platinum-sensitive indicator in advanced SOC (65). Moreover, the CTSF gene as a risk factor for resistance to a variety of tested drugs through drug susceptibility analysis, extensive resistance in the CTSF gene may be a potential reason for affecting disease outcome in patients with basal breast cancer, and selumetinib, SB590885, PLX4720, and Dabrafenib may be potential therapeutic or adjuvant therapeutic agents (66). Additionally, Shi (67) et al. screened 380 tumor stemness and immune (TSI)-related genes. Using a machine learning method, they constructed a five-gene TSI-specific signature (TSISig) comprising CPS1, CCR2, NT5E, ANLN, and ABCC2. This process involved integrating the tumor stemness index (based on mRNA expression, mRNAsi), immune score, mRNA expression profiles, and clinical data from the TCGA database. TSISig demonstrated robust prognostic predictive ability and served as an effective indicator for tumor recurrence and response to radiotherapy and immunotherapy in LUAD patients. Moreover, the stem cell index can also be used to identify potential therapeutic targets for CSCs by analyzing the gene expression patterns of stem cell-like tumor cells and identifying signaling pathways and proteins that are critical for the maintenance of stemness and tumorigenicity for the development of new drugs and therapies against CSCs. The mRNA expression-based stemness index (mRNAsi), which can represent degrees of dedifferentiation of HCC samples, was calculated by Feng (68)et al. to predict the drug response of sorafenib therapy and prognosis. Unsupervised cluster analysis was conducted to distinguish mRNAsi-based subgroups, and gene/gene set functional enrichment analysis was employed to identify key sorafenib resistance-related pathways. By analyzing the core regulatory genes of the PPAR signaling pathway, they identified four candidate target genes, (i) retinoid X receptor beta (RXRB), (ii) nuclear receptor subfamily 1 group H member 3(NR1H3), (iii) cytochrome P450 family 8 subfamily B member 1(CYP8B1), and (iv) stearoyl-CoA desaturase (SCD), as a signature to distinguish the response of sorafenib. They proposed and validated that the RXRB and NR1H3 could directly regulate NR1H3 and SCD, respectively. The results endorsed the combined use of SCD inhibitors and sorafenib as a promising therapeutic approach (68).

Finally, the stemness index has shown promise in identifying cancer patients who may benefit from targeted therapy or immunotherapy (42). In this regard, Wang (69) et al. retrieved gene expression data of 60 patients with gastrointestinal mesenchymal stromal tumor GIST from the Array Express database, applied CIBERSORT to calculate the immune infiltration level, used ssGSEA and ESTIMATE to calculate the cancer stemness index and the tissue purity, and implemented the connectivity map (CMAP) database to screen target drugs based on GIST’s CSC-like properties to screen targeted drugs. Consequently, the results suggested that there were differences in immune infiltration levels between metastatic and non-metastatic GIST groups and that low levels of T-cell infiltration were associated with high tumor purity and tumor stemness index, with correlation coefficients of -0.87 and -0.61 (p < 0.001), respectively (69). In addition, the cancer stemness index was positively correlated with cell purity (p < 0.001) and was higher in the metastatic group than in the non-metastatic group (p = 0.0017). Through the pharmacological mechanism of topoisomerase inhibitors, six molecular complexes may serve as the targets for GIST treatment (69). Studies for non-small cell lung cancer found that patients with a high stemness index responded better to immune checkpoint inhibitor therapy (PD-1/PD-L1 blockade) (70).

### 5.2 The stemness index serves as a potential biomarker for cancer progression and prognosis

Currently, the main factors affecting the prognosis of tumor patients include tumor grade, treatment modality, and independent prognostic influences. The stemness index has been investigated as a potential biomarker for predicting prognosis in patients with various types of cancers and identification of distant metastases in patients with high-risk cancers (57).

In a study of 355 breast cancer patients, the stemness index was found to be an independent predictor of distant metastasis-free survival and overall survival, suggesting that the stemness index can be used as a prognostic biomarker in breast cancer (71). Guo (72) analyzed gastric adenocarcinoma STAD cases in The Cancer Genome Atlas (TCGA) based on mRNAsi. mRNAsi analysis was performed on STAD by differential expression, survival analysis, clinical stage, and gender. Weighted gene co-expression network analysis (WGCNA) was used to identify useful modules and key genes, and enrichment analysis was carried out to annotate the functions and pathways of key genes. Finally, the expression levels of key genes in all the cancers were validated using the Gene Expression Omnibus (GEO) database in STAD, and the protein-protein interaction network was used to determine the relationship between the key genes. The results showed a decrease in mRNAsi scores with increasing tumor stage and T-stage, and a higher overall survival in highly grouped patients (72). Lyu (73) found that stemness index based on corrected mRNA expression was up-regulated in renal clear cell carcinoma (KIRC) tissues compared to non-tumor tissues and increased with tumor stage and grade. Similarly, EZH2 expression was associated with tumor-infiltrating immune cells, and epigallocatechin-3-gallate (EGCG) was determined to be the most potent inhibitor of EZH2. Notably, the percentage of FoxP3+ Treg cells in the peripheral blood mononuclear cells of ccRCC patients was significantly lower when cultured in spheroids pretreated with sunitinib, thus, Zhao (74) calculated mRNAsi from more than 500 lung adenocarcinoma patients from TCGA database based on a one-class logistic regression machine learning algorithm for pluripotent stem cells and their post-differentiation mRNA expression. mRNAsi-related key genes were identified by weighted correlation network analysis, and the results suggested that the mRNAsi was significantly higher in LUAD compared to normal lung tissues, whereby, patients with advanced LUAD demonstrated higher mRNAsi and poorer overall survival (OS). EZH2 was identified as a CSC marker and prognostic factor in KIRC patients. Huang (66) found that basal-like breast cancer carries the highest mRNAsi among all four breast cancer subtypes, and 385 mRNAsi-related genes were positively correlated with high mRNAsi values of basal breast cancer. High mRNAsi is closely associated with active cell cycle, DNA replication and metabolic reprogramming in basal-like breast cancer. Among them, TRIM59, SEPT3, RAD51AP1 and EXO1 can be used as independent protective factors, and CTSF and ABHD14B are used as risk factors, and the establishment of a prognostic model containing mRNAsi-related genes can effectively predict the survival in patients diagnosed with basal type breast cancer subtypes. Tan (75) classified gliomas into low-grade gliomas and glioblastomas based on mRNAsi-related genes by consensus clustering of TCGA dataset, and developed prognostic features related to stemness subtypes, which could effectively predict the prognosis in glioma patients. Tang (76)used single-sample gene set enrichment analysis (GSEA) to calculate the relative activities of the metabolic pathways in pancreatic ductal adenocarcinoma (PDAC) samples, and found that the overall survival (OS) of patients with high mRNAsi values was significantly lower than the patients with low mRNAsi values (P = 0.003). Moreover, weighted gene co-expression network analysis (WGCNA) revealed eight independent gene modules significantly associated with mRNAsi and 12 metabolic pathways, and two PDAC subgroups were identified based on unsupervised clustering of the key genes in each module, which demonstrated that PDAC samples with high mRNAsi values exhibited aberrant activation of multiple metabolic pathways, and the patients exhibited poor prognosis.

Overall, the AI-based Stem Cell Index stands as a powerful and evolving tool in cancer research with profound implications for understanding tumor grading, staging, and predicting prognosis (**Figure 5B**).The ongoing use, refinement, and integration of the AI-based Stem Cell Index in cancer research promises to revolutionize cancer treatment by offering more precise diagnostics, tailored treatments, and a deeper understanding of the intricate mechanisms governing tumor development and progression, thus, significantly improving patient outcomes and quality of life in cancer affected individuals.

### 5.3 Stemness index in tumor microenvironment and immune evasion

In the TME, a high stemness index is often associated with aggressive tumor behavior, therapy resistance, and poor prognosis. CSCs, which typically have high stemness scores, interact dynamically with components of the TME encompassing immune cells, stromal cells, and extracellular matrix (ECM) to maintain their stem-like state (77, 78) (**Figure 5C**). These interactions promote immune evasion by inducing immunosuppressive signaling pathways, modulating antigen presentation, and recruiting regulatory T cells and myeloid-derived suppressor cells. The TME, thus, becomes a sanctuary for CSCs, shielding them from immune surveillance and enhancing their survival (79, 80). Understanding the relationship between stemness and immune evasion offers valuable insights for therapeutic strategies aimed at disrupting CSC niches, reactivating anti-tumor immunity, and improving the efficacy of immunotherapies in cancers with high stemness signatures. Additionally, integrating stemness indices into prognostic models may aid in patient stratification and personalized treatment design (81).

### 5.4 Stemness index in single cell analysis and tumor evolution

In the context of single-cell analysis, the stemness index is particularly valuable for identifying subpopulations within heterogeneous tumor tissues. **Figure 5D** illustrates how the stemness index facilitates the identification of tumor subpopulations in heterogeneous tissues during single-cell analysis. By applying transcriptomic profiling at single-cell resolution, researchers can calculate stemness scores for individual tumor cells, uncovering gradients of differentiation and identifying (CSCs). These CSCs are often associated with therapy resistance, metastasis, and poor prognosis (82). Understanding the stemness index in single-cell data provides insights into tumor evolution, as cancer progresses through branching paths of clonal expansion, differentiation, and selection. Tumor cells with high stemness indices may serve as founders of new subclones, driving tumor heterogeneity and adaptive evolution under treatment pressure. Moreover, the spatial and temporal dynamics of stemness across a tumor can reveal how specific microenvironmental niches support CSC maintenance (50). Incorporating stemness indices into single-cell and spatial transcriptomics datasets helps reconstruct tumor lineage trajectories and evolutionary hierarchies (83, 84). Ultimately, this approach can inform therapeutic strategies by targeting CSC populations and their supporting environments, potentially improving long-term treatment outcomes by disrupting the cellular plasticity that fuels tumor progression and recurrence.

## 6 Cancer specific insights into applicability of the AI-driven methods in various cancer types

AI-driven stemness indices have been widely applied across various cancers to reveal prognostic patterns, immune landscapes, and therapeutic vulnerabilities, as shown in **Table 2**.

**Table 2:** Cancer specific insights into applicability of the AI-driven methods in various cancer type.

Cancer type	Application of stemness index	Key findings	AI/ML methods used	Notable biomarkers/pathways
NSCLC (Lung)	Classification into stemness subtypes; prognosis and immunotherapy prediction	High-stemness linked to poor survival and immune exclusion	LASSO, XGBoost, Random Forest	ARTN, CD8+ T-cell exclusion, EMT
HCC (Liver)	Prognostic modeling; immune profiling; methylation integration	High-stemness associated with low immune scores and worse prognosis	OCLR, LASSO, Multi-omics models	Wnt signaling, methylation patterns
CRC (Colon/Rectum)	Risk stratification and chemotherapy sensitivity	High-stemness tumors resistant to standard therapies	LASSO Cox, SVM	EMT, angiogenesis, tumor mutation burden
BLCA (Bladder)	Subtype identification and immune evasion prediction	High-stemness subtypes show immune suppression and worse outcome	Boruta, SVM, Consensus Clustering	TNFAIP6, immune checkpoints
PDAC (Pancreas)	Spatial distribution of CSCs and immune interaction analysis	AI revealed immune-suppressive niches near stem-like cells	Pathology AI + spatial transcriptomics	CD133+, CD8+ spatial mapping
GC (Gastric)	Drug sensitivity prediction and subtype classification	High-stemness linked to chemotherapy resistance	GSVA, XGBoost	Wnt/β-catenin, MAPK signaling
Pan-Cancer	Cross-cancer stemness comparison and biomarker discovery	Universal links between high stemness, immune evasion, and mutation load	Deep Learning, Multi-omics AI	Shared CSC markers, immune escape genes

### 6.1 Lung cancer – lung adenocarcinoma

In LUAD, the AI-derived mRNA stemness index (mRNAsi) is significantly higher in tumors than in normal lung tissue and increases with tumor stage. Patients with high mRNAsi had notably worse overall survival. A study combining OCLR-based mRNAsi with immune profiling identified a set of 144 “immune-stemness” genes. Hub genes including IL-6, FPR2, RLN3 were linked to poor prognosis and correlated with immune checkpoints and tumor mutational burden (TMB) (85).

### 6.2 Colorectal cancer

AI-based analysis stratified CRC patients into high- and low-mRNAsi groups. High-mRNAsi was associated with poorer overall survival in stage IV CRC, increased TMB, and altered immune infiltration patterns. Prognostic stemness signature: Weighted gene co-expression network analysis (WGCNA) and LASSO-Cox regression identified a three-gene prognostic signature (PARPBP, KNSTRN, KIF2C). This signature was validated via tissue immunofluorescence and incorporated into a nomogram outperforming TNM staging. High-stemness CRC tumors showed lower immune/stromal scores and reduced infiltration by macrophages, but higher CD8+ and T follicular helper cells, suggesting specific immune microenvironment remodeling (86).

### 6.3 Breast cancer

WGCNA of breast cancer transcriptomes linked mRNAsi to hub cell cycle genes (CDC20, PLK1, BUB1/BUB1B, NCAPG, KIF20A). These genes were overexpressed in advanced tumor stages and are promising therapeutic targets. Breast cancer stem cells (BCSCs) often display CD44+/CD24– phenotypes, undergo epithelial-mesenchymal transition (EMT), and are driven by Notch, HER2, and NF-κB signaling. Their metabolism supports self-renewal and therapy resistance (87).

### 6.4 Bladder, pancreatic, and gastric cancer

In bladder Cancer, AI-based clustering helps identify stemness-driven subtypes that are associated with poor prognosis and immune evasion. For example, TNFAIP6 was discovered as a critical gene in high-stemness tumors (88). AI combined with spatial pathology can reveal how cancer stem cells are arranged within the tumor and how they interact with the immune system (e.g., CD133+ cells co-located with immune suppression zones) in Pancreatic Ductal Adenocarcinoma (PDAC) (89). In Gastric Cancer (GC), stemness indices are used to stratify patients and predict drug sensitivity. Moreover, certain pathways (like Wnt signaling) are often enriched in high-stemness subtypes in GC (90).

### 6.5 Glioblastoma

While direct mRNAsi studies are limited, deep learning-based radiogenomic pipelines have been used to automatically segment tumors and predict survival in GBM. A 3D CNN radiomic signature yielded a C-index of 0.67 (vs. 0.64 for traditional methods), with significant patient stratification (91). Wnt/β-catenin stemness signaling: Preclinical work highlights Wnt pathway activation in glioblastoma stem cells (GSCs), with elevated β-catenin, TCF/LEF1, LGR5, and c-Myc—suggesting a possible basis for integrating AI-based signaling pathway quantification (92).

## 7 Challenges and limitations

Despite the promising advances in applying artificial intelligence (AI) to develop stemness indices in cancer, several challenges and limitations remain, which need to be addressed before these tools can be fully integrated into clinical practice.

One major challenge is the biological complexity of cancer stemness. Cancer stem cells (CSCs) are highly heterogeneous, existing in dynamic states that vary not only between tumor types but also within a single tumor. Capturing this heterogeneity through AI models is difficult, particularly when using bulk transcriptomic data that averages signals from diverse cell populations. This can obscure important variations, limiting the accuracy of stemness indices (93). Another significant limitation is the lack of standardized frameworks for defining and measuring stemness. Different studies use varying gene sets, computational methods, and cutoffs to calculate stemness scores, leading to inconsistent results that are hard to compare or reproduce. This heterogeneity complicates efforts to validate stemness indices across cohorts and cancer types (94). Data-related issues also pose challenges. Heterogeneity across datasets, including differences in sequencing platforms, sample processing, and patient demographics, can introduce biases. Many AI models are trained on retrospective public datasets such as TCGA, which may not represent the diversity of clinical populations, affecting the generalizability of findings (95).

Another critical limitation is the lack of prospective clinical validation. Most studies rely on retrospective analyses, and few have demonstrated how AI-based stemness indices perform in predicting patient outcomes or guiding therapy decisions in real-time clinical settings. Moreover, overfitting remains a concern, especially with complex machine learning models applied to relatively small datasets. This can lead to overly optimistic performance metrics that do not hold up in independent validation. Finally, integrating AI-derived stemness indices with existing clinical workflows requires models to be interpretable and explainable, yet many AI methods remain “black boxes,” which limits clinician trust and adoption. Addressing these challenges through rigorous methodological standardization, large-scale prospective studies, and explainable AI will be essential for translating stemness indices into practical cancer care tools (96).

While stemness indices such as mRNAsi and mDNAsi have demonstrated promising applications in cancer research, several studies have reported contradictory or limited findings. For instance, the predictive value of stemness scores varies across tumor types; in some cancers, high stemness correlates with poor prognosis, while in others, no significant association is observed (97). Additionally, discrepancies arise when comparing indices derived from different data platforms (e.g., TCGA vs. GEO), often due to batch effects and data normalization inconsistencies. Some AI-based models show reduced reproducibility when applied to external validation cohorts, highlighting concerns about overfitting and lack of generalizability (98). Moreover, stemness scores sometimes fail to reflect the functional heterogeneity of cancer stem cells (CSCs) within the tumor microenvironment. These inconsistencies underscore the need for standardized methodologies, cross-cohort validation, and integration of multi-omics data to improve the reliability of stemness-based metrics in cancer biology.

## 8 Future directions

The application of artificial intelligence (AI) to quantify cancer stemness is a rapidly evolving field with substantial promise, but several key areas warrant further development to maximize clinical impact. One important future direction is the integration of spatial transcriptomics and pathology-based AI. By combining gene expression data with spatial localization of cells within the tumor microenvironment (TME), researchers can gain a more nuanced understanding of how cancer stem cells (CSCs) interact with immune and stromal cells, potentially revealing new therapeutic targets. Another promising avenue is the advancement of single-cell stemness models. Current AI-based stemness indices primarily rely on bulk tumor data, which can obscure heterogeneity. Single-cell technologies, coupled with machine learning, will allow more precise identification of CSC subpopulations and their dynamic states, improving prognostic accuracy and therapy stratification. Furthermore, the transition from retrospective computational analyses to prospective clinical trials is essential. Validating AI-derived stemness scores in real-world patient cohorts will help establish their utility in guiding treatment decisions and predicting outcomes. Developing pan-cancer deep learning frameworks that integrate multi-omics data across tumor types can uncover universal and cancer-specific stemness signatures, enhancing personalized medicine. Finally, synergizing AI with mechanistic biology is crucial for drug discovery. Understanding the molecular underpinnings of AI-identified stemness features will facilitate development of novel therapies targeting CSCs, addressing therapy resistance and relapse. Overall, these directions emphasize a multidisciplinary approach to fully harness AI’s potential in stemness research and precision oncology.

Collectively, these challenges highlight the need to refine stemness modeling—potentially through transfer learning, data augmentation, multi-omics integration, and single-cell approaches—to enhance its reliability and clinical applicability, particularly in the context of rare tumors.

## 9 Conclusions

The stemness index, constructed on the basis of artificial intelligence, is a measure of the extent of stem cell-like features in cancer cells that has recently emerged as a promising biomarker for identifying different cancer subtypes, thus, aiding in prognostication and therapeutic decision-making in breast, colorectal, lung, hepatocellular carcinoma, and glioblastoma. However, its clinical utility encounters various challenges and limitations that are required to be addressed for enhanced applicability in cancer management. Most importantly, lack of standardized methods for calculating the Stemness Index is one of the major challenges, leading towards variability and potential biases in results across studies and datasets. Therefore, establishing standardized protocols is crucial for ensuring consistency and comparability of the results, thus obtained. Additionally, limited availability of high-quality datasets, especially for rare or less common cancer subtypes is a significant constraint. Since training and validation of the Stemness Index models require diverse and comprehensive datasets, the scarcity of such datasets for less prevalent cancers hinders the accuracy and generalizability of the Index. While promising, the clinical utility and reliability of the cancer stemness indices need rigorous validation through large-scale prospective studies. Validating its effectiveness in predicting prognosis, treatment response, and guiding therapeutic decisions in diverse patient populations is essential for its implementation in a clinical setting. Various factors, including TME, patient demographics, and comorbidities, could influence the applicability of the Stemness Index. Therefore, it is critically important to apprehend and account for the confounder to ensure the accuracy and reliability of the stemness index in reflecting accurate stemness characteristics in various cancer subtypes. Nonetheless, additional research studies are highly necessitated to overcome existing challenges and address the aforementioned limitations, and to establish the clinical relevance and utility of the stemness index as a putative biomarker for various cancer subtypes, at large.
